# First step toward gene expression data integration: transcriptomic data acquisition with COMMAND>_

**DOI:** 10.1186/s12859-019-2643-6

**Published:** 2019-01-28

**Authors:** Marco Moretto, Paolo Sonego, Ana B. Villaseñor-Altamirano, Kristof Engelen

**Affiliations:** 1 Unit of Computational Biology, Research and Innovation Centre, Fondazione Edmund Mach, via E. Mach 1, 38010 San Michele all’Adige, Italy; 2 Laboratorio Internacional de Investigación Sobre el Genoma Humano, Universidad Nacional Autónoma De México, 76230 Juriquilla, Querétaro, Mexico; 3 Centro de Ciencias Genómicas, Universidad Nacional Autónoma de México, 62210 Cuernavaca, Morelos Mexico

**Keywords:** Transcriptomic, Gene expression, Microarray, Rna-seq, Compendia, Data integration

## Abstract

**Background:**

Exploring cellular responses to stimuli using extensive gene expression profiles has become a routine procedure performed on a daily basis. Raw and processed data from these studies are available on public databases but the opportunity to fully exploit such rich datasets is limited due to the large heterogeneity of data formats. In recent years, several approaches have been proposed to effectively integrate gene expression data for analysis and exploration at a broader level. Despite the different goals and approaches towards gene expression data integration, the first step is common to any proposed method: data acquisition. Although it is seemingly straightforward to extract valuable information from a set of downloaded files, things can rapidly get complicated, especially as the number of experiments grows. Transcriptomic datasets are deposited in public databases with little regard to data format and thus retrieving raw data might become a challenging task. While for RNA-seq experiments such problem is partially mitigated by the fact that raw reads are generally available on databases such as the NCBI SRA, for microarray experiments standards are not equally well established, or enforced during submission, and thus a multitude of data formats has emerged.

**Results:**

COMMAND>_ is a specialized tool meant to simplify gene expression data acquisition. It is a flexible multi-user web-application that allows users to search and download gene expression experiments, extract only the relevant information from experiment files, re-annotate microarray platforms, and present data in a simple and coherent data model for subsequent analysis.

**Conclusions:**

COMMAND>_ facilitates the creation of local datasets of gene expression data coming from both microarray and RNA-seq experiments and may be a more efficient tool to build integrated gene expression compendia. COMMAND>_ is free and open-source software, including publicly available tutorials and documentation.

## Background

Transcriptomic studies started over 20 years ago with the first spotted microarray [1] while the first RNA-seq experiments appeared about a decade ago [2–4]. Since then the number of transcriptomic experiments performed has constantly grown, favoured, among other things, by the increase of technical quality and the decreasing prices [5]. Nowadays large studies profiling expression of genes and their association with several experimental conditions are commonplace, and the wealth of public information is a huge help for scientific investigation. Nevertheless, most of the true potential for reuse and integration remains untapped because of the vast heterogeneity of such datasets and the difficulties in combining them. With the advent of systems biology, data integration emerged as a prevailing aspect to take full advantage of such rich sources of information [6]. Several approaches have been proposed to fulfill the need to effectively integrate gene expression data and they can generally be categorized as being either direct integration or meta-analysis. The former directly consider the sample-level measurements within each study, and merge these into a single data set [7]. Meta-analysis, on the other hand, integrates gene expression analysis combining information from primary statistics (such as *p*-values) or secondary statistics (such as lists of differentially expressed genes) resulting from single studies. Those studies combine the information from several data sources defining confidence levels subjectively for each individual study without a general scheme. Meta-analysis is a common method to integrate conclusions from different studies [8].

Both approaches have been widely adopted and many tools have been developed to exploit or further analyse such datasets [9–13]. Regardless of the strategy used to combine and analyse a large amount of gene expression experiments, the first step in common with all these approaches is the acquisition of raw data. COMMAND>_ (COMpendia MANagement Desktop) is a web application developed in order to facilitate the creation and maintenance of local collection of gene expression data and have been successfully used to build gene expression compendia such as COLOMBOS [14] and VESPUCCI [15]. It has been designed with flexibility in mind in order to deal with the disparate ways in which gene expression data are published, and to be easily extended to deal with new technologies.

## Implementation

**Fig. 1:**
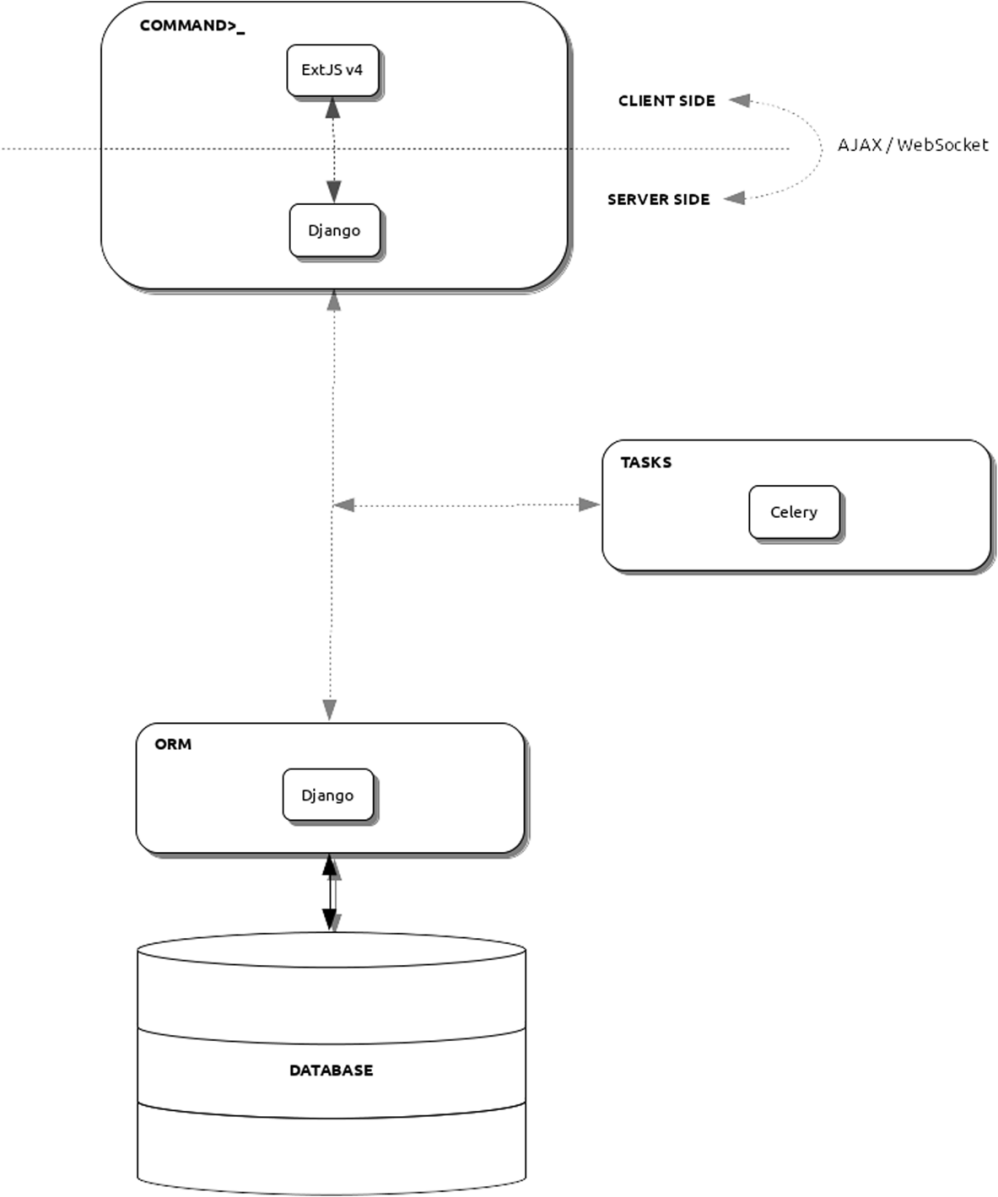
COMMAND>_ infrastructure. The Graphical User Interface (GUI), on the client-side, is developed using the ExtJS Javascript framework that communicates using both AJAX calls and WebSocket to the server-side part of the application developed using the Django framework. All the business-logic has been developed in Python within the Django framework that is in charge of managing the database connection with the Object Relational Mapping (ORM) layer. Celery is the task queue distributed system used to run and manage tasks

COMMAND>_is a multi-user web application developed in Python 3 using the Django 1.11 framework for the backend; the web interface has been developed using ExtJS 6.2 with a look and feel typical of desktop applications (Fig. 1). Despite being developed as a single page application, it allows users to navigate using browser buttons. By default it relies on PostgreSQL as Database Management System (DBMS), but the Django Object Relational Mapping (ORM) allows it be used with other DBMSs as well. COMMAND>_ uses both AJAX and WebSocket (via Django Channel) for client-server communications. WebSocket ensures a two-way communication between the web interface and a Python backend, easing the problem of continuously polling the server for updates on time-consuming tasks. Intensive tasks such as downloading and parsing files are managed asynchronously by the Celery task queue system so that many processes can run simultaneously (8 by default). COMMAND>_ is a complex application with several layers that work together. To ease the deployment process we provide a Docker Compose file, thus having a working instance is just a matter of running one configuration file. Since COMMAND>_ relies on several third-party software, performance depends in part on the specific software requirements. The default Python scripts are designed to keep the memory footprint as low as possible and scale linearly with respect to the input size, because many of them might run concurrently. The complete requirements list is available at the documentation page. COMMAND>_ has been designed to be adapted to different gene expression platforms and currently handles platforms of two kinds, microarray and RNA-seq, but can be extended to allow for more platforms to be managed. Gene expression data itself are modeled as one possible type of data that can be collected. By extending specific classes, as reported in the online documentation, COMMAND>_ can be adapted to potentially handle any kind of quantitative data.

### Data model

**Fig. 2:**
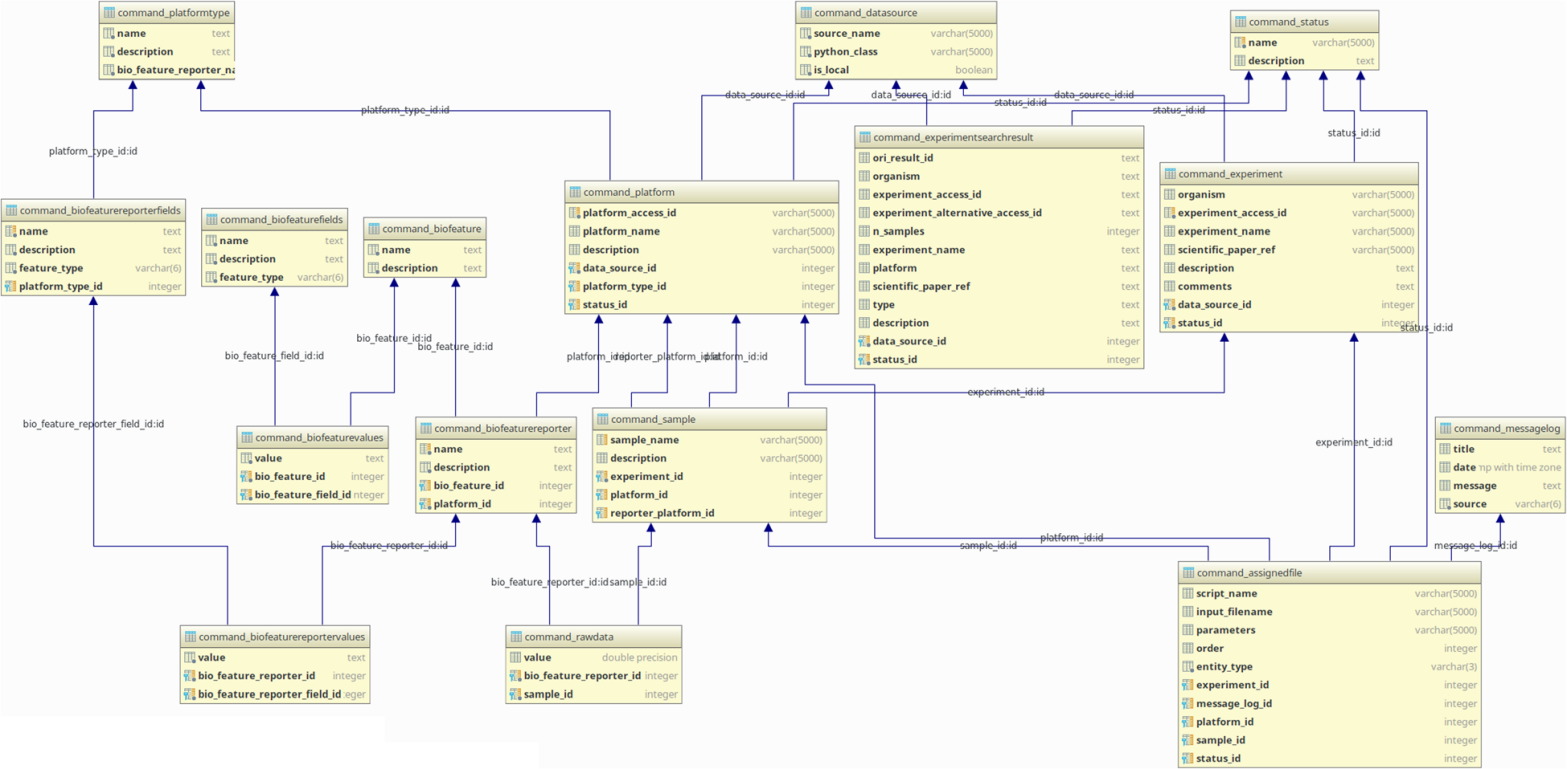
The database schema that represents the data model. The core part is represented by the *sample* table that holds the information of the *experiment* it belongs to, the *platform* used to measure it as well as the link to the *raw_data* measurements

The basic concept behind the data model and how it is implemented in the database (Fig. 2) revolves around the idea that a set of measurements for several biological features (such as genes in case of gene expression data) are collected across different samples. The collected values might be direct or indirect measurements of such biological features and depends on the type of platform used in the experiment. In case of microarrays for example, each measurement refers to a single probe (a *reporter* in the data model) and thus it is an indirect measurement of gene activity. Samples can then be thought as a set of *reporter* measurements taken with a *platform* that is therefore a set of reporters. Biological features (as genes) and reporters (as probes) might have different properties (fields) such as name and sequence that can be used to couple the two entities. The three entities *experiment*, *platform*, and *sample* as well as *biological features* and *reporters* also hold meta-data, such as original ids, names and descriptions.

## Results and discussion

### Workflow

**Fig. 3:**
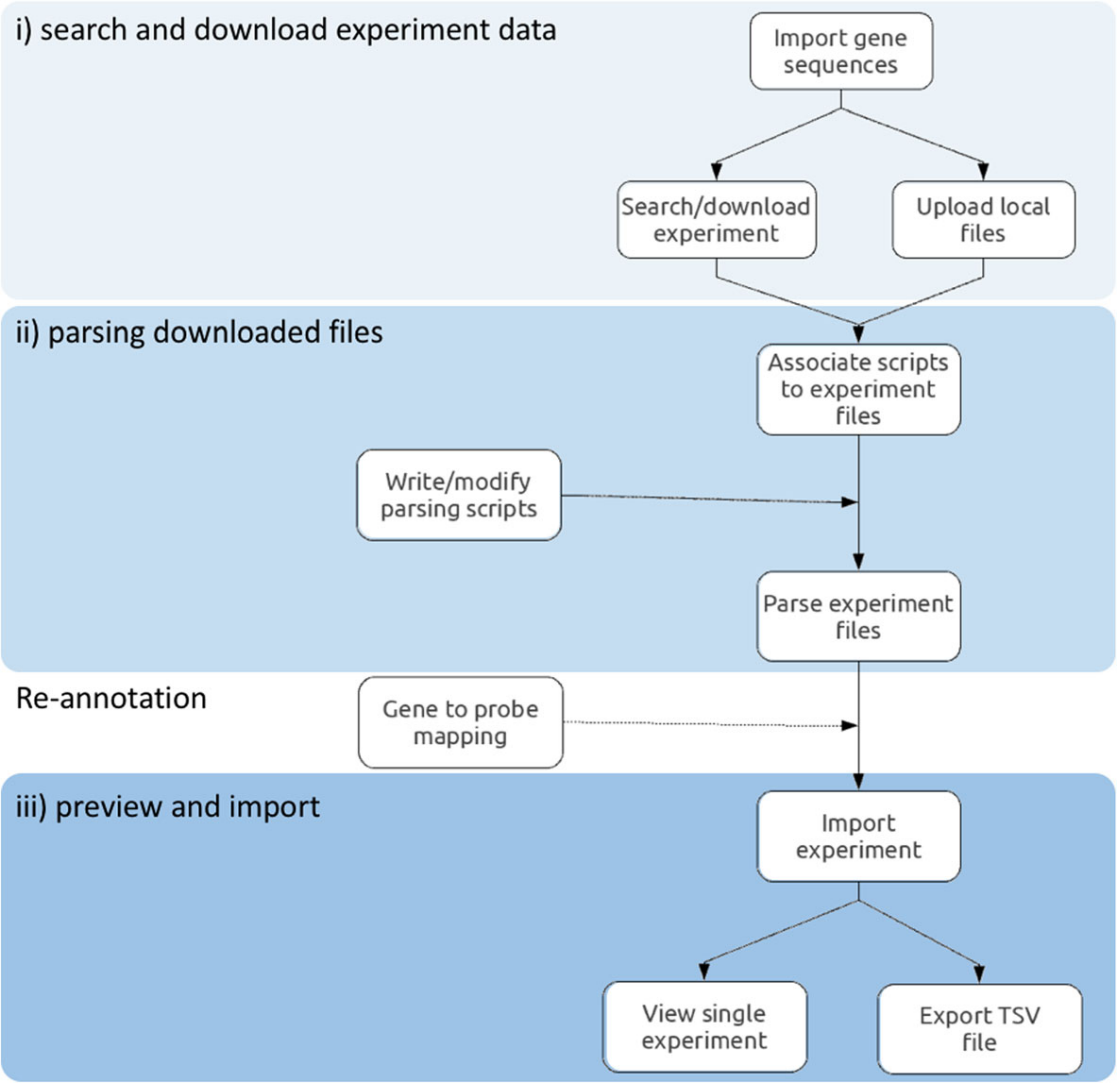
The flowchart of the typical workflow. Users start by searching and downloading experiments from public databases or uploading files for local experiments. Experiment files are then associated with parsing scripts and the parsing phase runs in background. Once experiment is parsed can be imported into the database and, if necessary, probes can be mapped to genes

COMMAND>_ is a multi-user application. From the web interface it is possible to create users and groups and grant privileges. Admin users have unlimited access, while normal users might be limited to work only on specific compendia and/or with a subset of functionalities. The typical workflow can be divided into three steps: i) search and download experiment data, ii) parsing downloaded files, iii) preview and import experiment data into the local database (Fig. 3).

**Fig. 4:**
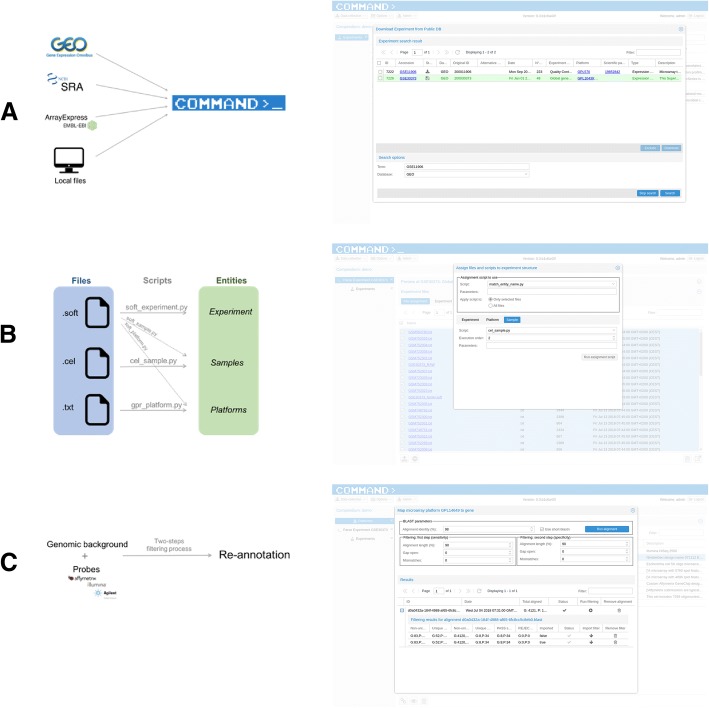
**a** The download experiment dialog. From this dialog it is possible to search experiment from GEO, SRA or ArrayExpress and download all the related files. **b** The file assignment dialog. From this dialog users assign scripts to experiment files in order to parse the information. **c** Probe to gene mapping dialog. This dialog allows users to perform the alignment and the two-step filtering used to re-annotate microarrays by mapping probes to genes

A mandatory prerequisite for being able to perform these steps is to first establish the genomic background for the expression data, by uploading a FASTA file with gene sequences. Users can then import experiments starting by searching and downloading them from public databases or uploading local files (Fig. 4a). The supported databases are (at the moment) NCBI GEO [16], SRA [17] and EBI ArrayExpress [18]. Once the search has been performed, users can select one or more experiments and start the download process. Compressed files will be automatically extracted in a temporary folder.

The pivotal point is the assignment of downloaded files together with parsing scripts to entities (*experiment*, *platforms* and *samples*) to mine only the relevant information (Fig. 4b). The scripts can be created or modified directly within the interface and are responsible for parsing input files and populating each part of the data model, i.e. measurement data and meta-data for *experiment*, *platforms* and *samples*. Once scripts are assigned to downloaded files, they can run independently and the results can be inspected using the preview interface. If the experiment appears to be complete, it can be imported into the database. Any possible error that might occur during parsing or importing of the experiment will be reported in the system log.

**Fig. 5:**
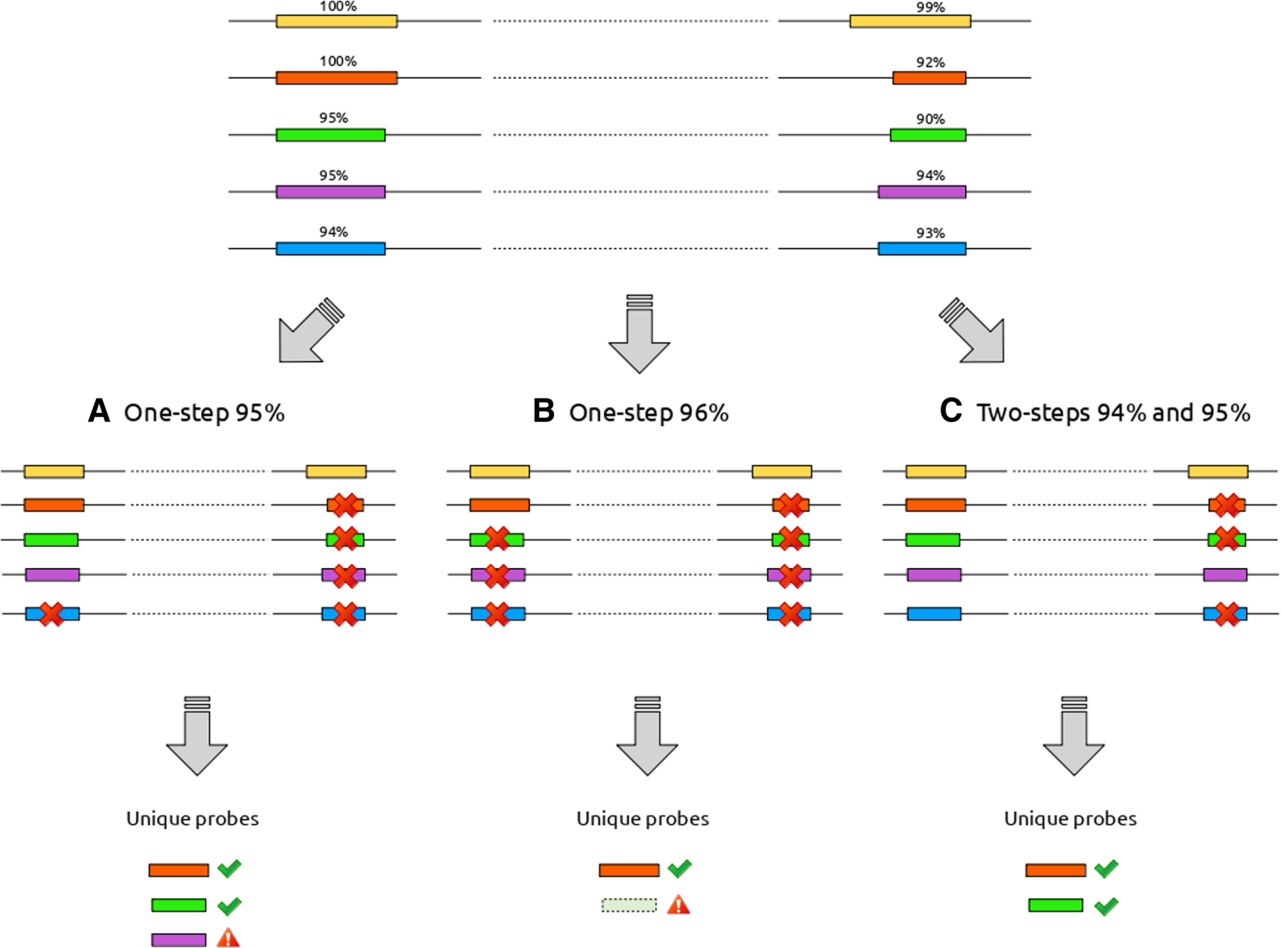
Advantages of two-step filtering. The five probe sequences (yellow, orange, green, purple and blue) all aligns twice with different scores. Starting from the assumptions that, for this specific example case, a reasonable threshold is 95% or more and that for a sequence to be considered aligned uniquely the score difference between the alignments of the same probe should be more than 3%, we consider three different scenarios. The leftmost one (scenario **a**) presents the case of a single-step filtering with a threshold equal to 95%. In this case the probe selected as unique ones are the orange, green and purple one. While the first two probes are compliant with our assumptions, the purple one should be discarded since it aligns twice with a score of 95 and 94% respectively. To avoid this situation we might try do adjust the threshold raising it to 96% as depicted in the scenario **b**. The problem in this case is given by the fact that we would miss the green probe. Using a two-step filtering, as shown in scenario **c**, avoids both this unwanted situations since the first filter (at 94%) would retain both the purple probe alignments (that will be discarded since the alignment is not unique) and the blue probe that will then be removed by the second filter at 95% leaving only the orange and the green probes as expected

When a new microarray platform gets imported, it would be necessary to map its probes to genes. The probe to gene mapping is a fundamental process carried out performing a BLAST+ [19] alignment and a two-step filtering (Fig. 4c). The alignment might take a while for platforms with a lot of probes, especially when using the short-blastn option, and the result cannot be used as-is for the probe to gene mapping. Bad alignments need to be filtered out in order to retain only the most plausible ones, i.e. the alignments that most likely represent the “true” mRNA-to-probe hybridization process. The filtering step is usually fast and can be performed several times on the same alignment result to test different threshold choices. The two-steps filtering tries to mitigate the side effect of a simpler filtering (Fig. 5) and it is performed to guarantee that probes map to genes with high similarity (restrictive alignment threshold), while also mapping unambiguously to a unique position avoiding cross-hybridization issues in the measurements (less restrictive alignment threshold). Since probes coming from different microarrays generally differ in terms of length, origin, and sequence quality, parameters and cut-off thresholds can be adjusted in order to always obtain the reasonably best possible results according to each platform’s specific characteristics and user needs. The probe to gene mapping step has the advantage of enhancing data homogeneity since all microarray platforms will be annotated using the same gene list (i.e. the same genomic background represented by the FASTA file with gene sequences uploaded during the initial step). Moreover, annotating the microarray with the latest available data is often preferable since it might improve the expression data interpretation [20, 21]. If probe sequences are not available, or relying on the default annotation is more appropriate, it is possible to manually associate probes to genes using, for example, the manufacturer annotation (gene identifiers). All the parameters and re-annotation are stored on COMMAND>_, so that the procedure is completely reproducible.

In case of RNA-seq platforms, these steps are not necessary since the imported measurements (raw counts) are directly related to genes of the defined genomic background without the need for reporters like probes as for microarray experiments. Once FASTQ files are downloaded and associated to samples, the user will need to create the index file for the genomic background to be used in the alignment program. A FASTA file with gene sequences imported by the user would be automatically created and put in the experiment directory to be used as target for the index creation script. By default, FASTQ files will be then trimmed using Trimmomatic [22] and expression level quantified using Kallisto [23]. Users that wish to use different programs, could copy them to the COMMAND>_ directory and to write a Python wrapper script to use them.

The three steps described in the workflow are specific for gene expression data, but would be the same in case of other kind of quantitative data. For example, exon or small-RNA sequencing could easily be used by adopting a different genomic background, thus uploading a FASTA file with exons or small-RNAs sequences respectively. The importance of the genomic background definition lies in the fact that it establishes exactly what is measured by the imported experiments. Considering that the genomic background should not change during data collection, not all quantitative data are equally suitable for being imported in COMMAND>_. For example, metagenomic experiments for which Operational Taxonomic Units (OTUs) change from sample to sample (and even more from experiment to experiment) would not be an ideal type of quantitative data to be collected.

### Comparison with similar tools

To the best of our knowledge there are no other tools that offer all the options COMMAND>_ does. Nevertheless, we will report the main differences between COMMAND>_ and other similar tools (see Table 1). GEOquery [24] is a package written for the R programming language (http://www.R-project.org/) that allow R users to easily connect, retrieve, parse and extract expression data from GEO ready to be used in downstream analysis. The ArrayExpress [25] R package works similarly to GEOquery but for the ArrayExpress database. GEOmetadb [26] and SRAdb [27] both allow the user to query GEO and SRA within the R environment, but they require to download an SQLite file that contains the totality of GEO/SRA metadata. Compendiumdb [28] is an R package framework used to parse and store expression information into a relational database that can be queried from within the R environment for subsequent analysis. VirtualArray [29] is another R package used to combine raw data from diverse microarray samples (or experiments) and generates a combined object for further analysis. It also implements several batch effect removal methods but it is not available for the latest Bioconductor version. Microarray retriever [30] is a web-application used to query and download expression data from both GEO and ArrayExpress, but is currently unavailable.

**Table 1:** Functionalities comparison between COMMAND>_ and other tools used to collect gene expression data from public databases

Tool	R	Local	GUI	GEO	AE	SRA	DB	ANN	Search	Note
COMMAND>_	NO	YES	YES	YES	YES	YES	YES	YES	YES	
GEOquery	YES	NO	NO	YES	NO	NO	NO	NO	NO	
ArrayExpress	YES	NO	NO	NO	YES	NO	NO	NO	YES	
GEOmetadb	YES	NO	NO	YES	NO	YES	NO	NO	YES	requires the dowload of an sqlite file with meta information
SRAdb	YES	NO	NO	YES	NO	YES	NO	NO	YES	requires the dowload of an sqlite file with meta information
compendiumdb	YES	NO	NO	YES	NO	NO	YES	NO	NO	
virtualArray	YES	YES	NO	YES	YES	NO	NO	NO	NO	allow to normalize data and correct for batch effect, available only for Bioconductor <= 2.14
Microarray retriever	NO	NO	YES	YES	YES	NO	YES	NO	YES	unavailable

Each column represent one functionality, respectively: R (the program is an R package), local (the program allow to use local data), GUI (the program provides a Graphical User Interface), GEO (the program connects to GEO), AE (the program connects to ArrayExpress), SRA (the program connects to SRA), DB (the program provides a database to store expression data), ANN (the program allows to annotate probes), Search (the program allow to perform queries using free text besides accession id) and Note (the program has special features or limitations)

The main difference between all these tools, except for Microarray retriever, and COMMAND>_ is that all of them are Bioconductor packages and run within the R programming environment. The advantages are that Bioconductor is a strong and reliable environment and different packages can be used in combination to perform a vast amount of different analysis. Despite being a great tool for data analysis, R and Bioconductor are not meant for data retrieval, and management of large amount of data can be problematic since R programs, without specific packages such as parallel, are by default single-threaded process, the data are completely stored in RAM and thus don’t easily scale to handle large datasets.

COMMAND>_ has been developed with the specific goal of simplifying this part. It relies on a relational database and a task queue system such as PostgreSQL and Celery respectively to easily scale when number of experiments grows significantly. In this regard they might be thought more as complementary tools with R to be used to analyse the datasets collected using COMMAND>_. In COMMAND>_ many operations can be done using the Graphical User Interface (GUI) such as the re-annotation tool which allows the user to produce an optimized annotation instead of relying on default ones. It is important to highlight that the re-annotation step allows perfect reproducibility of the analysis since all parameters are stored within COMMAND>_. Finally, despite being a graphical tool offering a friendly user experience, COMMAND>_ gives the same flexibility of a command-line environment to manage all possible situations through its Python editor.

### Case study

In order to demonstrate COMMAND>_ functionalities, we present several case studies available within the on-line documentation. Moreover, we used it here for searching, downloading, parsing, re-annotating and exporting a collection of small airway samples from patients affected by Chronic Obstructive Pulmonary Disease (COPD) [31]. The original study is a collection of 273 samples from three Affymetrix microarray experiments retrieved from the Gene Expression Omnibus (GEO): GSE8545, GSE20257 and GSE11906. We start retrieving the GEO experiments used in the study using the “Download Experiment From Public Database” dialog with the GSE Series ID as term and GEO as database (Fig. 4a). Before starting to parse the experiments we need to import the gene sequences to be used for the probe mapping step. The parsing procedure starts by selecting one experiment and pressing the “Parse/Import experiment” button. The parsing interface is divided into three collapsible sections: the top one shows the experiment data preview, the middle one contains the experiment files browser and the assignment tool used to couple parsing scripts and experiment files, while the bottom section is the Python editor. Having the original probes is highly encouraged in order to take advantage of the probe to gene mapping functionality. Since probe sequences are not included in this experiment, we have to download them separately from the Affymetrix Support site and upload them into COMMAND>_ using the “Upload file” button. Once all files are in place we are ready to start assigning parsing scripts to the experiment files. Since we don’t need to change any information related to the experiment entity we will start with platform-related files, i.e. HGU133Plus2_Hs_ENSG_probe_tab. The assignment procedure is itself based on the execution of a Python script, and in this way we can automatically assign a vast amount of files using user-defined rules. For this specific case we will tell COMMAND>_ to parse the “HGU133Plus2_Hs_ENSG_probe_tab” file using the “gpr_platform.py” script. To correctly parse the platform file we have to inform the “gpr_platform.py” about the field names to be used for the probe id and the probe sequence. The sample files assignment will proceed similarly by selecting all CEL files (we can use the filter by file names) and giving “cel_sample.py” as script to be used. This time we will use the “match_entity_name.py” assignment script in order to have COMMAND>_ to automatically couple CEL files with the corresponding samples (Fig. 4b). The last file to be assigned is the soft file that contains the meta-data for all entities, experiment, platform and samples. Once again we use the “assign_all.py” to assign “soft_experiment.py”, “soft_platform.py” and “soft_sample.py” scripts to experiment, platform and samples respectively. After inspecting that all the assignments are correctly done we are ready to run the parsing scripts. Once the parsing is done we can inspect the results in the “Preview” section and import the experiment. We will have to repeat the same procedure for the remaining experiments.

Once that all the raw data are imported into the database we can map the probes for the GPL570 platform to the human genes we already imported. This fundamental step consists in two parts, the alignment and filtering of alignment results. For the alignment step we can chose a quite stringent identity threshold (such as 95% or 98%) since both probes and genes belong to the same species. The two-step filtering thresholds are set to 95% alignment length, 0 gap and 3 mismatches for the sensitivity step and 98% alignment length, 0 gap and 1 mismatch for the specificity step (Fig. 4c). The chosen threshold captures the idea that probes might align (even if not perfectly) on more than one gene resulting in an unusable probe, and, require a higher minimum alignment quality for a probe to be considered reliable. As stated previously this wouldn’t be possible using only a single filter (Fig. 5). In this specific case choosing different thresholds will result in little differences since probes and genes come from the same organism. This step is increasingly relevant when more and more probes are designed for a different organism than the one we are using as the genomic background and for which we have the gene sequences, such as might be the case for different strains of bacteria or different cultivars of plant crops.

After the alignment process is complete, we can set the filtering parameters and run the filtering. Once the filtering is done, we are able to import the probe to gene mapping. Finally, we can export the resulting raw data in both TSV and HDF5 file format.

## Conclusion

In this paper we present COMMAND>_, a web-based application used to download, collect and manage gene expression data from public databases. COMMAND>_ relies on a DBMS for data persistence and a set of customizable Python scripts to extract only relevant information from public gene expression databases. COMMAND>_ is a multi-user application that allow teamwork via definition of groups of users with specific privileges on each of the defined gene expression compendia. Moreover, it eases the long-time maintenance of such gene expression compendia storing a system log with all the relevant information about the operations performed. COMMAND>_ is a tool in constant development with new features to be added with newer versions. It is easily extendable to readily manage new technology platforms as they appear, for new data formats to be parsed, and even for new quantitative data types to be imported. This is reflected in the software architecture as well as in the data model.

## Availability and requirements

Project name: COMMAND>_.

Project home page: https://github.com/marcomoretto/command

Operating systems: any supporting Docker Compose or Python 3 (tested on Linux).

Programming languages: Python, Javascript.

Other requirements: full requirements list available at https://raw.githubusercontent.com/marcomoretto/command/master/requirements.txt

License: GNU GPL v3.

Any restrictions to use by non-academics: none.

## Abbreviations

COMMAND>_: COMpendia MANagement Desktop
COPD: Chronic Obstructive Pulmonary Disease
DBMS: DataBase Management System
GUI: Graphical User Interface
ORM: Object Relational Mapping
OTU: Operational Taxonomic Unit
